# Quality of life of patients with metastatic pancreatic adenocarcinoma initiating first-line chemotherapy in routine practice

**DOI:** 10.1186/s12904-020-00610-4

**Published:** 2020-07-10

**Authors:** Berta Laquente, Teresa Macarulla, Cristina Bugés, Marta Martín, Carlos García, Carles Pericay, Sandra Merino, Laura Visa, Teresa Martín, Manuela Pedraza, Beatriz Carnero, Raquel Guardeño, Helena Verdaguer, Alejandro Mut, David Vilanova, Adelaida García

**Affiliations:** 1 Institut Catala d’Oncologia, Hospital Duran i Reynals, Hospitalet de Llobregat, Avda. De la Gran Via, 199, 08908 L’Hospitalet de Llobregat, Barcelona, Spain; 2 Vall d’Hebrón University Hospital and Vall d’Hebrón Institute of Oncology (VHIO), Barcelona, Spain; 3 Institut Català d’Oncologia (ICO) – Hospital Germans Trias i Pujol, Badalona, Spain; 4 Hospital de Sant Pau, Barcelona, Spain; 5 Complejo Hospitalario de Burgos, Burgos, Spain; 6 Corporació Sanitaria Parc Taulí, Sabadell, Spain; 7 Hospital Sant Joan de Reus, Reus, Spain; 8 Hospital del Mar, Barcelona, Spain; 9 Hospital Universitario de Salamanca, Salamanca, Spain; 10 Complejo Asistencial Universitario de León, León, Spain; 11 Hospital El Bierzo, Ponferrada, Spain; 12 Institut Català d’Oncologia (ICO), Girona, Spain; 13 Celgene S.L.U, Madrid, Spain

**Keywords:** Pancreatic Cancer, Health-related quality of life, Cancer chemotherapy, Performance status, EORTC QLQ-C30

## Abstract

**Background:**

Despite advances in surgery, radiotherapy, and chemotherapy, pancreatic adenocarcinoma often progresses rapidly and causes death. The physical decline of these patients is expected to impact their quality of life (QoL). Therefore, in addition to objective measures of effectiveness, the evaluation of health-related QoL should be considered a matter of major concern when assessing therapy outcomes.

**Methods:**

Observational, prospective, multicenter study including patients with metastatic pancreatic adenocarcinoma who started first-line chemotherapy in 12 Spanish centers. Treatment and clinical characteristics were recorded at baseline. Patients’ health-related quality of life, ECOG, and Karnofsky index were measured at baseline, at Days 15 and 30, and every four weeks up to 6 months of chemotherapy. Health-related quality of life was measured using the EORTC-QLQ-C30 and EQ-5D questionnaires. Other endpoints included overall survival and progression-free survival.

**Results:**

The study sample included 116 patients (median age of 65 years). Mean (SD) scores for the QLQ-C30 global health status scale showed a significant increasing trend throughout the treatment (*p* = 0.005). Patients with either a Karnofsky index of 70–80 or ECOG 2 showed greater improvement in the QLQ-C30 global health status score than the corresponding groups with better performance status (*p* ≤ 0.010). Pain, appetite, sleep disturbance, nausea, and constipation significantly improved throughout the treatment (*p* < 0.005). Patients with QLQ-C30 global health status scores ≥50 at baseline had significantly greater overall survival and progression-free survival (*p* = 0.005 and *p* = 0.021, respectively). No significant associations were observed regarding the EQ-5D score.

**Conclusions:**

Most metastatic pancreatic adenocarcinoma patients receiving first-line chemotherapy showed an increase in health-related quality of life scores throughout the treatment. Patients with lower performance status and health-related quality of life at baseline tended to greater improvement. The EORTC QLQ-C30 scale allowed us to measure the health-related quality of life of metastatic pancreatic adenocarcinoma patients receiving first-line chemotherapy.

## Background

Despite recent advances in chemotherapy treatments, metastatic pancreatic adenocarcinoma (mPAC) remains incurable and survival rates are still low [1]. In the absence of treatments achieving long-term survival, chemotherapy aims to slow tumor progression and relieve symptoms [2, 3]. However, mPAC is an aggressive disease, and the burden of physical symptoms, together with the adverse effects of chemotherapy, lead to patients’ rapid physical decline and the subsequent deterioration of their quality of life (QoL) [2, 3]. In this scenario, health-related quality of life (HRQoL) becomes a significant concern in the management of patients with mPAC [4, 5].

Given the acknowledged importance of HRQoL, several clinical trials have reported HRQoL and other QoL-related outcomes in mPAC patients [5, 6]. Typically, trials including mPAC report a remarkable burden of toxicities associated with chemotherapy regimens. Nevertheless, some of these trials provide supportive evidence that favor chemotherapy in terms of HRQoL, particularly in patients who responded to it [3, 6]. In line with these observations, global HRQoL and HRQoL-related outcomes, such as pain and fatigue, were associated with survival in the clinical trial setting [7–10]. However, the methodologies used to evaluate HRQoL—usually introduced as secondary endpoints—were heterogeneous, thus they precluded comparisons between studies, often leading to inconsistent results [5, 6]. Furthermore, thorough selection criteria from randomized controlled trials (e.g., patients with relatively good physical status) are unlikely to reflect a real-life practice scenario. To date, real-world data is limited to a few studies proving the association between HRQoL and survival [11–15], and the positive impact of nab-paclitaxel plus gemcitabine treatment in HRQoL of patients with partial response or stable disease [16].

The American Society of Clinical Oncology (ASCO) defines clinically meaningful outcomes in the clinical trial setting as a balance between toxicity and efficacy, stressing the importance of HRQoL measures as indicators of toxicity [17]. Similarly, real-life studies recommend routine assessments of HRQoL to guide treatment decisions [18]. In this observational prospective study, we describe the evolution of HRQoL in patients with mPAC treated with first-line chemotherapy in routine clinical practice, and analyze patients’ clinical characteristics that may influence their HRQoL.

## Methods

### Study design and population

This was an observational, prospective, multicenter study including patients with histologically confirmed mPAC (either recurrent or de novo) from 12 Spanish centers, who had started first-line chemotherapy. Adult patients (i.e., aged ≥18 years) with a Karnofsky Index (KI) ≥70 and a life expectancy ≥6 months, who attended routine follow-up visits between October 2014 and October 2015, were consecutively included in the study. No other recruitment sources were considered. Patients that were pregnant or breastfeeding, participating in a clinical trial, or unable to understand or answer questions related to their health were excluded from the study. Baseline demographic and clinical data were collected from the medical records at the initiation visit (i.e., at study entry, before starting the first-line treatment). Assessments, which were scheduled to match routine visits, were performed at baseline, at Days 15 and 30, and every 4 weeks up to 6 months after starting chemotherapy (Treatment Period). After this time, patients were followed up every 6 months for up to 18 months to assess survival (Follow-Up Period).

### Endpoints, variables and assessments

The primary endpoint was the evolution of HRQoL in patients with mPAC treated with first-line chemotherapy in a routine clinical practice setting. HRQoL was assessed using two self-administered questionnaires: the European Organization for Research and Treatment of Cancer (EORTC) Quality of Life Questionnaire (QLQ-C30) (Version 3.0), and the European Quality of Life-5 Dimensions (EQ-5D). The QLQ-C30 is a 30-item, cancer-specific multidimensional questionnaire designed for prospective clinical studies that includes five functional scales (role, physical, emotional, cognitive and social functioning), three symptom scales (fatigue, pain, and nausea and vomiting), a global health and quality-of-life scale, additional specific symptoms commonly reported by cancer patients (dyspnea, loss of appetite, sleep disturbance, constipation, and diarrhea), and the perceived financial impact of the disease and treatment [19]. The EQ-5D is a 5-item generic measure of health status, including five dimensions (mobility, self-care, usual activities, pain/discomfort and anxiety/depression) and a Visual Analog Scale (VAS) [20]. Both questionnaires, which have been translated to Spanish and validated in the Spanish population [21, 22], were administered during routine follow-up visits at baseline and at each visit during the Treatment Period.

Baseline demographic characteristics considered in the study included age, sex, and weight loss in the last 3 months. Clinical and treatment characteristics included relevant concomitant diseases, date of diagnosis of the primary tumor, location of the metastatic disease, previous adjuvant treatments for non-metastatic disease (if any)—regimen, number of cycles, start and completion dates, and response—, and first-line treatment prescribed (schedule and initiation date). Together with the administration of HRQoL questionnaires, routine assessments were performed at baseline and during the 6-month Treatment Period, including blood tests, biochemistry, tumor evaluation, performance status (PS) (ECOG and KI), treatment modification, and concomitant medication. Other variables were reasons for discontinuation of chemotherapy, recorded during the Treatment and Follow-Up Periods, dose modifications and adverse events, recorded during the Treatment Period. Adverse events (AE) were classified according to the Common Terminology Criteria for Adverse Events (CTC-AE, version 4.02) of the National Cancer Institute (NCI) (http://ctep.cancer.gov/reporting/ctc.html).

### Statistical analysis

Categorical variables and outcomes were presented as frequencies and percentages, whereas continuous variables and outcomes were presented as mean and standard deviation (SD), or as median and interquartile range (Q1,Q3). Categorical outcomes were compared using the Chi-Square test or the Fisher’s exact test. Correspondingly, continuous outcomes were compared using the T-Test, the ANOVA, or their non-parametric counterparts Wilcoxon or Kruskal-Wallis tests. OS and PFS curves were plotted using the Kaplan-Meier estimator, and compared using the log-rank test. Survival analyses are described by median and 95% confidence interval (CI). The improvement or deterioration in HRQoL was defined as an increase or decrease of ≥5 points in the EORTC QLQ-C30 global health status score with respect to baseline [23]. Overall changes in the score throughout the study visits were assessed using the adjusted linear mixed model. The significance level for all analyses was set at a two-sided α = 0.05.

To estimate the minimum sample size needed, we assumed a rate of QoL improvement of 5%. The rate of QoL improvement was defined as a minimum increase of 5–10 points in EORTC QLQ-C30 global health status scores with respect to baseline. Based on this assumption, a sample size of 110 patients was deemed necessary to estimate the proportion of patients with an improvement in QoL 6 months after starting first-line chemotherapy with a ± 4.1% precision and a 95% CI. Presuming that 10% of patients may not complete the EORTC QLQ-C30, the approximate number of patients included in the study was 120. All analyses were performed using the statistical SAS software for Windows (version 9.4).

## Results

### Patient characteristics and treatment outcome

Of the 120 patients recruited, four were excluded for not having a first-line chemotherapy regimen scheduled (*n* = 1), being enrolled in a clinical trial (*n* = 1), declining to participate in the study for personal reasons after signing the informed consent (*n* = 1), and withdrawing for not having started treatment (*n* = 1). The resulting study sample included 116 patients, of which 101 (87.1%) had concomitant diseases, with a median (Q1, Q3) age of 65.0 (59.5, 71.5) years and a median (Q1, Q3) Body Mass Index (BMI) of 23.9 (22.2, 26.6) kg/m^2^. Table 1 summarizes the demographic and clinical characteristics of study patients at baseline and main treatment characteristics.

**Table 1:** Characteristics of study patients and treatment

Demographic characteristics	
Sex, *n (%)* (*n* = 116)	
Male	70 (60.3)
Females	46 (39.7)
**Clinical characteristics**	
Diagnosis, *n (%)* (*n* = 116)	
Metastatic after relapse/progression	25 (21.6)
De novo metastatic	91 (78.4)
% Weight loss in the last 3 mo (median, Q1, Q3) (*n* = 74)	9.2 (4.5, 14.1)
Weight loss > 10%, *n (%)*	33 (44.6)
CA 19.9 UI/mL (median, Q1, Q3) (*n* = 90)	725.7 (83.0, 7323.0)
Comorbidities, *n (%)* (*n* = 116)	
Hypertension	48 (41.4)
Diabetes	35 (30.2)
Performance Status	
ECOG Performance Status, *n (%)* (*n* = 103)	
0–1	84 (81.6)
2	19 (18.4)
Karnofsky Index, *n (%)* (*n* = 115)	
90–100	60 (52.2)
70–80	55 (47.8)
**Treatment characteristics and outcome**	
Treatment, *n (%)* (*n* = 113)	
Gemcitabine + nab-paclitaxel	73 (64.6)
Gemcitabine monotherapy	21 (18.6)
FOLFIRINOX or mFOLFIRINOX	14 (12.4)
Other combinations	5 (4.4)
Treatment schedule, *n (%)* (*n* = 101)	
Patients with any change in the dose or schedule	57 (56.4)
Patients with any delay in the treatment	59 (58.4)
Reasons for treatment discontinuation, *n (%)* (*n* = 111)^a^	
Progression	60 (54.1)
Toxicity	15 (13.5)
Clinical deterioration	15 (13.5)
Lost to follow-up	1 (0.9)
Voluntary withdrawal of the study	1 (0.9)
Death	3 (2.7)
Other reasons	19 (17.1)
Best response obtained in the first-line treatment, *n (%)* (*n* = 87)	
Complete response	3 (3.4)
Partial response	24 (27.6)
Stable disease	32 (36.8)
Progression	28 (32.2)

^a^Patients could have more than one reason for treatment discontinuation

Of these 116 patients, 113 (97.4%) started chemotherapy in the study setting and were treated for a median (Q1, Q3) of 3.9 (1.4, 6.7) months. Median OS was 9.0 months (95% CI 6.5–11.1) and median PFS was 6.0 months (95% CI 4.6–7.8). ORR was 32.6%, with three (3.4%) and 24 (27.6%) patients achieving complete and partial responses, respectively; 32 patients (36.8%) had stable disease. The number of patients assessed gradually declined during the Treatment Period due to death or disease progression (and subsequent start of second-line regimens), with 40 (34.2%) study patients remaining at the end of the 6-month treatment, and 22 (19.0%) completing all study visits.

Overall, 112 patients (96.6%) had 962 adverse events (AEs), of which 111 were serious, 390 were related to treatment, and 171 were of grade 3–5. The most frequent grade 3–5 AEs were neutropenia (32 events), asthenia (14 events), thrombocytopenia (7 events), pneumonia (6 events), pyrexia (6 events), febrile neutropenia (6 events), diarrhea (5 events), and anemia (5 events). Table 2 summarizes the frequency of most common treatment-related grade ≥ 3 AEs, classified by System Organ Class.

**Table 2:** Common treatment-related adverse events (> 1% of patients) of grade ≥ 3 classified by System Organ Class and Preferred Term, (*n* = 113)

	No. (%)
**General disorders and alterations related to the administration site**	
Asthenia	6 (5.3)
**Hematological disorders**	
Neutropenia	21 (18.6)
Febrile neutropenia	5 (4.4)
Thrombocytopenia	5 (4.4)
Anemia	2 (1.8)
Hemato-toxicity	2 (1.8)
**Gastrointestinal disorders**	
Diarrhea	3 (2.7)
**Nervous system disorders**	
Neurotoxicity	2 (1.8)

### Evolution of HRQoL

**Fig. 1:**
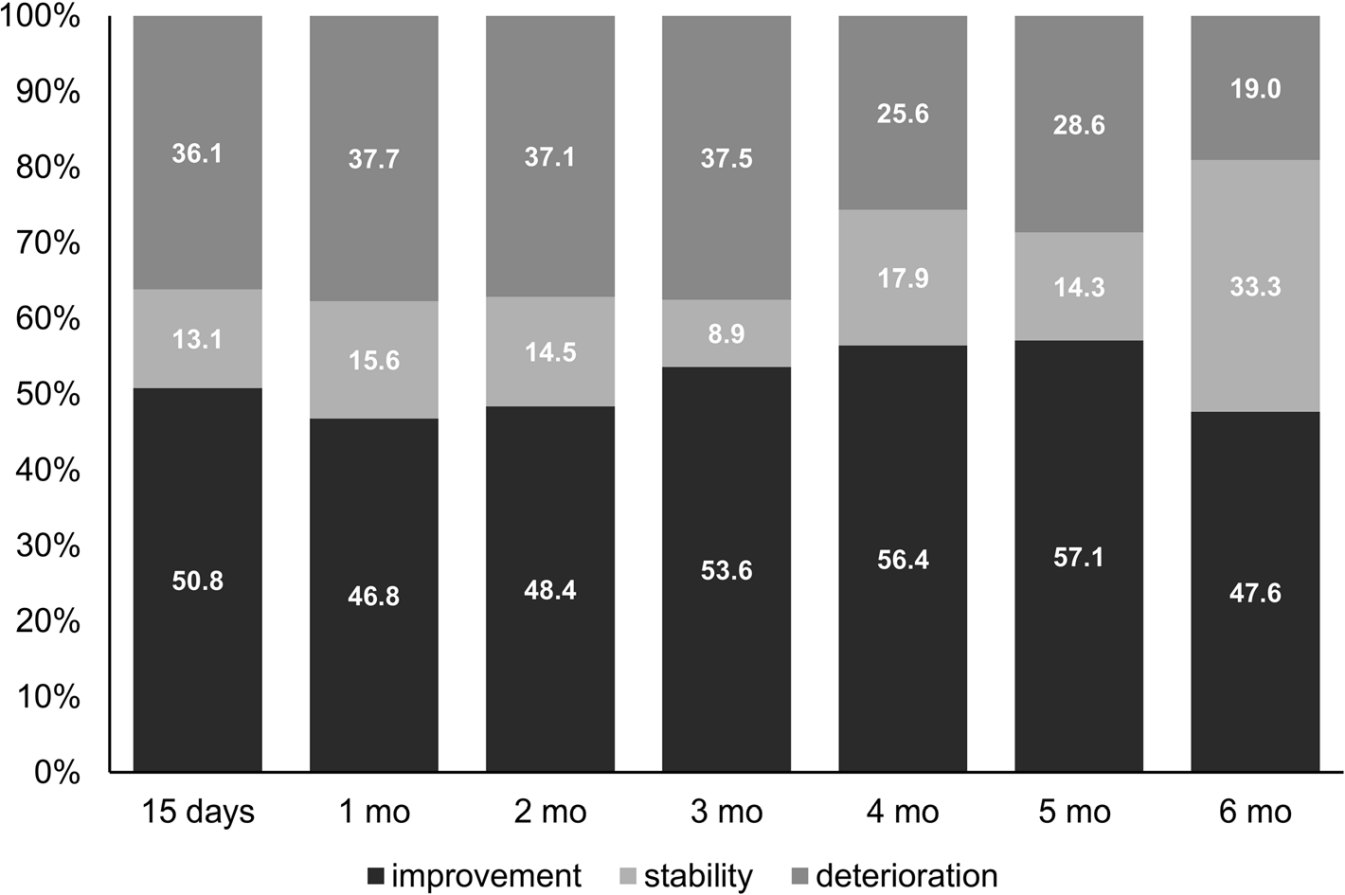
Qualitative changes in QLQ-C30 global health status scale scores at each of the study visits throughout the treatment period. The number of patients experiencing improvement, stability or deterioration in their HRQoL is presented as a percentage over the total of patients attending each of the visits

The adjusted linear mixed model revealed a significant difference in QLQ-C30 global health status scores throughout the Treatment Period (*p* = 0.005), with scores showing an increasing trend at each visit. Overall, mean (SD) scores increased from 53.7 (24.6) at baseline to 66.7 (18.1) at the 6-month visit. Furthermore, at each of the follow-up visits, the percentage of patients with better QLQ-C30 global health status scores than at baseline was ≥46.8% (Fig. 1). Fifty-seven (65.5%) patients improved their HRQoL in at least one visit, 15 (17.2%) showed a decline or a stable score, and 15 (17.2%) showed a decline in all visits. Of the 15 patients showing only deterioration in HRQoL compared to baseline, 13 (86.7%) had QLQ-C30 global health status scores ≥50 at baseline. Overall, HRQoL deterioration was more frequent in patients with higher QLQ-C30 global health status scores at baseline: the percentage of patients with deterioration at each study visit ranged from 24 to 50% and from 6 to 15% in patients with baseline QLQ-C30 global health status scores ≥50 and < 50, respectively (*p* < 0.0001). EQ-5D (dimensions and VAS) did not change significantly throughout the Treatment Period.

**Fig. 2:**
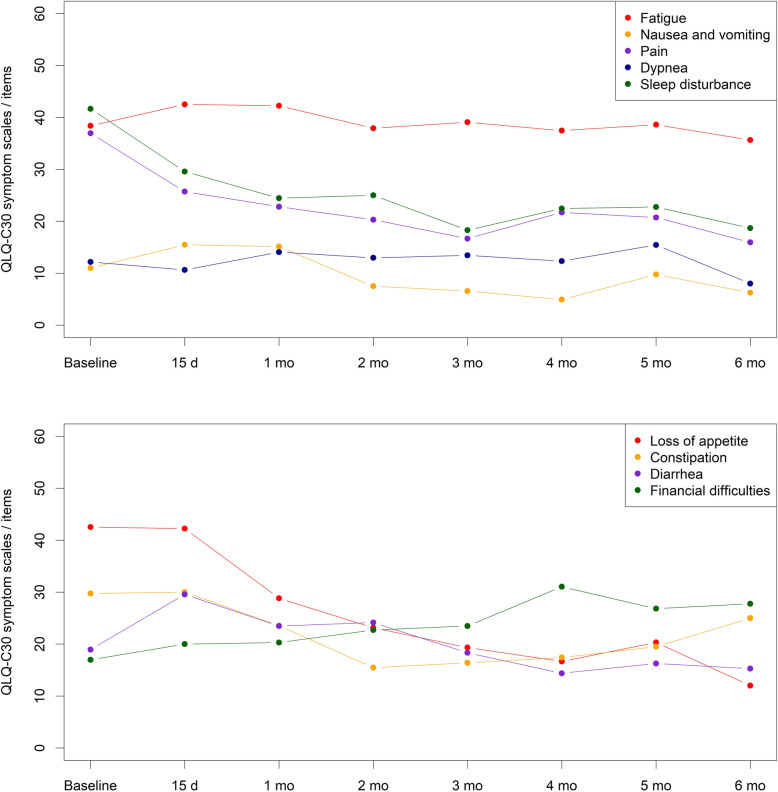
Evolution of the mean EORTC QLQ-C30 scores for fatigue, nausea, pain, dyspnea and sleep disturbance (A); and appetite loss, constipation, diarrhea and financial impact (B) throughout the treatment period

Regarding the symptom scales included in the QLQ-C30, the ones assessing pain, appetite, and sleep disturbance improved throughout the treatment, as shown by a significant decrease in their mean scores (Fig. 2). Mean scores for nausea and constipation also decreased, albeit modestly, whereas mean scores for fatigue, dyspnea, diarrhea and financial impact did not show statistically significant differences throughout the Treatment Period (*p* = 0.63, *p* = 0.64, *p* = 0.06, and *p* = 0.22, respectively).

**Fig. 3:**
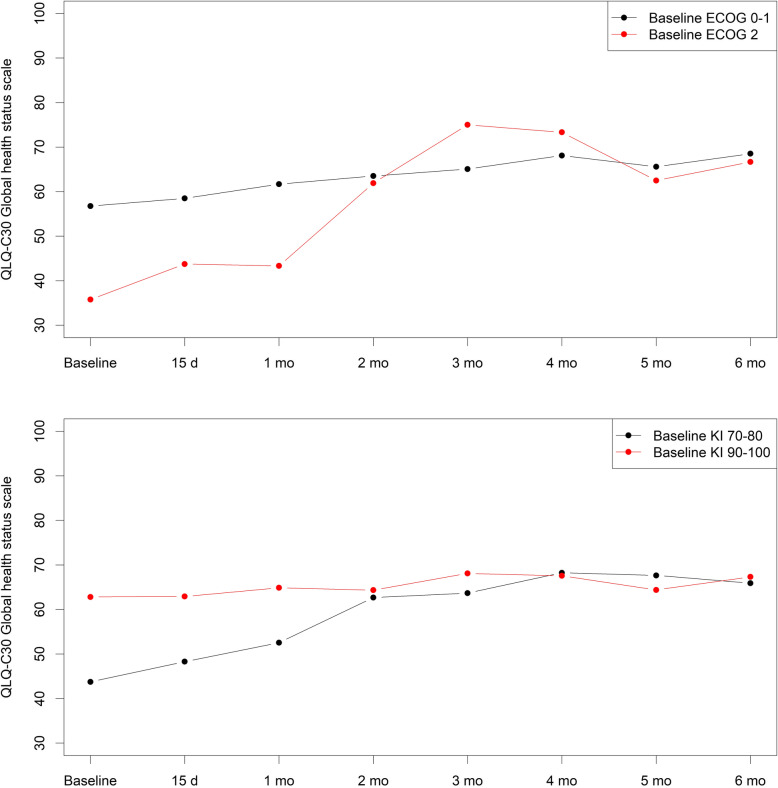
Evolution of the mean QLQ-C30 global health status scale scores according to the baseline Karnosfky Index (A) and ECOG (B) throughout the treatment period

**Fig. 4:**
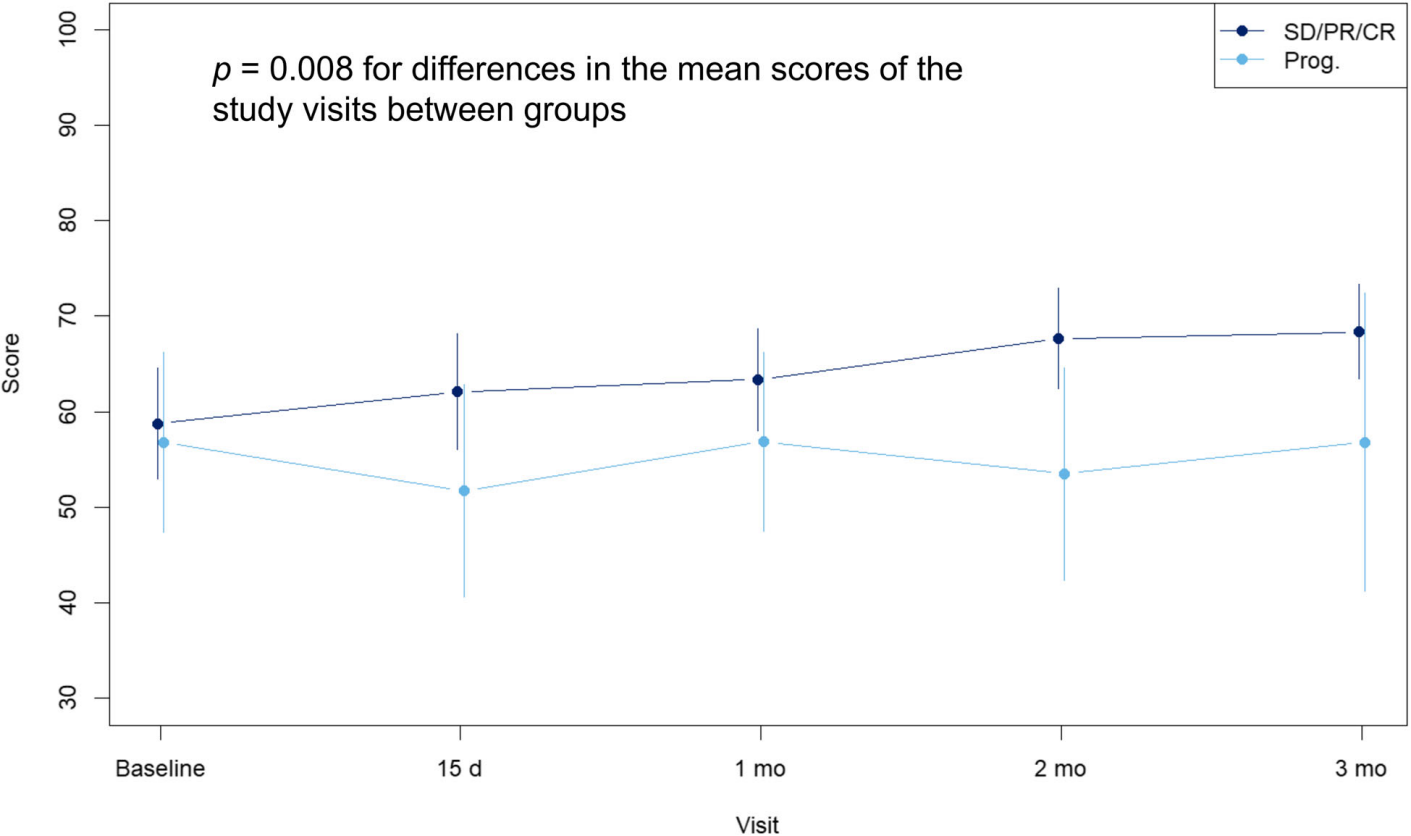
Evolution of the mean QLQ-C30 global health status scale scores according to the best response to first-line treatment. No evaluable patients remained in the progression group beyond month 3 of treatment. **SD:** stable disease, **PR:** partial response, **CR:** complete response

Of all variables analyzed for their influence on HRQoL, baseline PS and treatment response significantly influenced QLQ-C30 global health status scores. Patients with either KI of 70–80 or ECOG 2 at baseline showed a significantly greater improvement in HRQoL than the corresponding groups with better PS, reaching similar QLQ-C30 global health status scores after 2 months of chemotherapy (Fig. 3). Regarding treatment response, baseline QLQ-C30 global health status scores were similar in patients whose best response during first-line treatment was either partial/complete or stable disease and in patients with progression as best response. However, patients whose best response was partial/complete or stable disease had significantly higher QLQ-C30 global health status scores at each study visit during the first 3 months than those with progression as best response (Fig. 4). No significant associations were observed with EQ-5D scores.

### Relationship between health status measures and survival

**Fig. 5:**
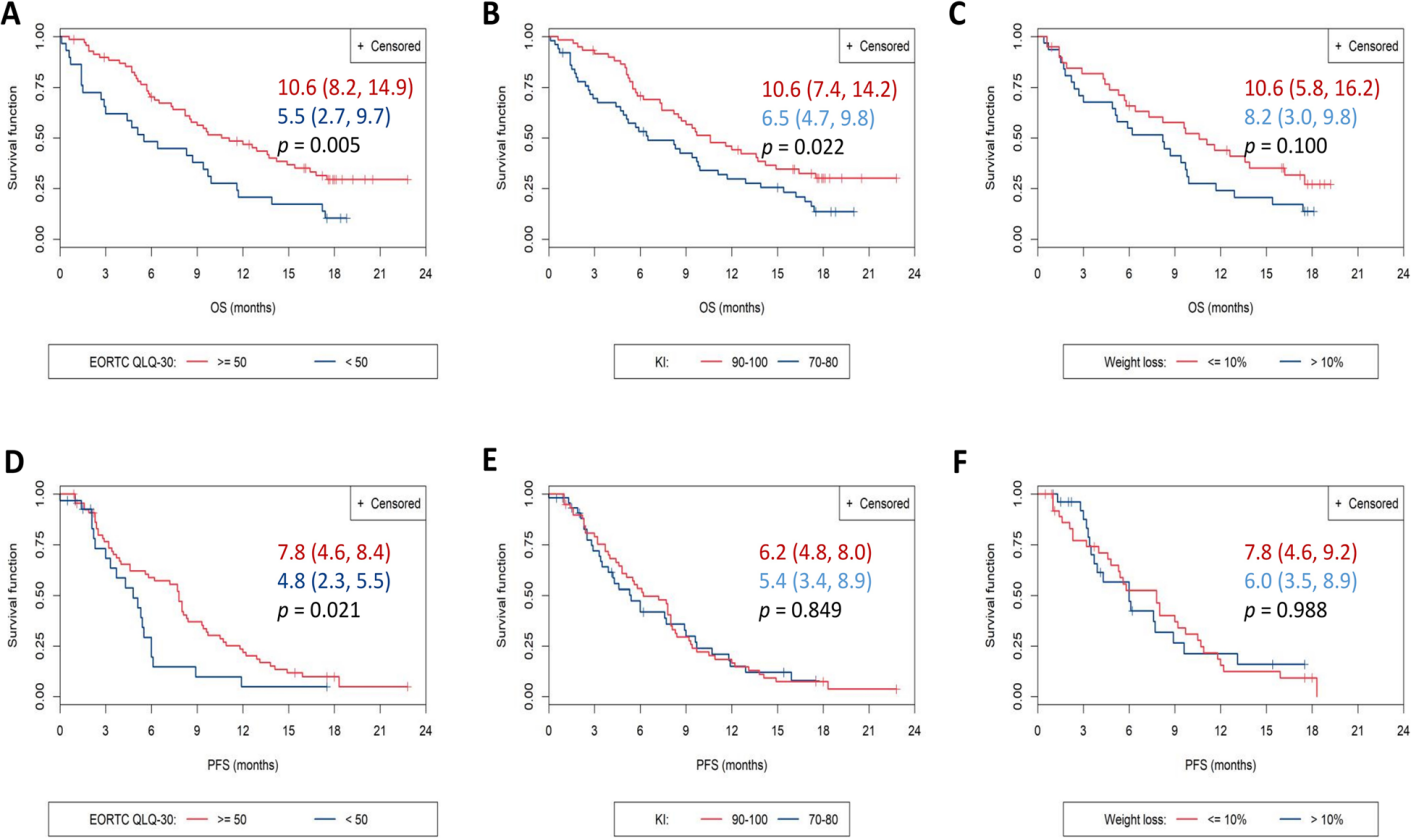
Overall survival (A,B,C) and progression-free survival (D,E,F) depending on baseline EORTC QLQ-C30 global score (A,D), baseline Karnofsky Index (B,E), and weight loss in the previous 3 months (C,F). Survival is presented as median (95% CI); *p*-values correspond to the Log-rank test for inter-curve differences

Of all baseline parameters analyzed for their potential role as prognosis factors for survival, KI and QLQ-C30 global health status scores showed a significant association with OS and/or PFS (Fig. 5). Patients with baseline KI of 90–100 had a significantly higher OS (but not a higher PFS) than patients with baseline KI of 70–80. Patients with baseline QLQ-C30 global health status scores ≥50 had a significantly higher OS and PFS than those scoring < 50. Weight loss in the previous 3 months (> 10% vs. < 10%) did not have a significant influence on OS and PFS.

## Discussion

In this observational prospective study, we found that patients receiving any first-line chemotherapy treatment in routine clinical practice experienced positive changes in various health domains, resulting in an improvement of the HRQoL; this improvement was more notorious in patients with poorer HRQoL at treatment start ―therefore, with most room for improvement. Mean scores of the QLQ-C30 global scale and pain, appetite, sleep disturbance, nausea, and constipation symptom scales improved throughout the chemotherapy treatment, whereas scores from the EQ-5D questionnaire (dimensions and VAS) remained unchanged. The evolution of HRQoL throughout the first-line treatment, measured using the QLQ-C30 global health status score, was significantly influenced by patients’ PS and treatment response. Baseline QLQ-C30 global health status scores influenced OS and PFS, suggesting a prognostic value for this factor.

In this study, two instruments were used to measure HRQoL: the EORTC QLQ-C30 scale and the EQ-5D. While QLQ-C30 showed good responsiveness and was sensitive to changes in HRQoL throughout the Treatment Period, EQ-5D (questionnaire and VAS) did not reflect any changes in HRQoL. This finding is in line with a previous observational study in patients with mPAC, in which QLQ-C30, but not EQ-5D, enabled to identify changes in HRQoL [16]. In contrast, another clinical trial reported significant changes in the scores of pain symptoms using EQ-5D in patients with advanced pancreatic cancer [24]. To our knowledge, very few studies on mPAC patients used EQ-5D to assess HRQoL, likely because other cancer-specific tools, such as QLQ-C30 and cancer site-specific instruments, are available. In fact, the National Cancer Institute of Canada Clinical Trial Group chose QLQ-C30 as a standard questionnaire in clinical trials [25]. Even though EQ-5D lacked sensitivity, its response rates were slightly higher than those of the QLQ-C30 questionnaire: mean response rate throughout study visits was 79.0 and 76.1% for EQ-5D dimensions and VAS, respectively, and 70.3% for the complete QLQ-C30 questionnaire. The EORTC QLQ-C30 global health status score, used to investigate correlations with baseline PS, treatment response and prognosis, reached a mean response rate of 78.6% throughout the study. While a consensus on acceptable response rates for patient-reported outcomes remains unestablished, a 70–80% response rate can be considered reasonable to good [26]. Although assessing the validity of these questionnaires was out of the scope of our study, the sensitivity observed with the QLQ-C30 questionnaire suggests it is suitable for measuring changes in HRQoL of mPAC patients undergoing first-line chemotherapy.

Despite the importance of assessing HRQoL to evaluate the balance between toxicity and effectivity of chemotherapy, studies focusing on changes in HRQoL of mPAC patients treated with chemotherapy are scarce, heterogeneous, and have often reported conflicting results. Kristensen et al. systematically reviewed 23 clinical trials in advanced pancreatic cancer, which included the assessment of HRQoL as a secondary endpoint. Of the 14 studies reporting changes in HRQoL compared to baseline, five observed an improvement in at least one treatment arm, three observed worsening in at least one treatment arm, and the remaining seven reported no change [6]. Our results, showing a statistically significant 13-point increase in mean QLQ-C30 global health status scores from baseline to Month 6, are in line with the five studies reporting an improvement in HRQoL in the trial setting [7, 9, 27–29]. Likewise, the decrease in pain scores during the Treatment Period in our cohort, which indicates an improvement of this symptom, are in line with the seven studies (out of eight clinical trials reporting on the evolution of pain scores) showing an improvement in pain [9, 28, 30–34]. Even though the mean QLQ-C30 global health status score improved in our study population throughout the Treatment Period, 17% of patients showed only deterioration. Interestingly, patients with worse HRQoL at baseline were more likely to show an improvement throughout the Treatment Period than those with better HRQoL at baseline.

Asides from assessing the evolution of HRQoL in real-life patients receiving first-line chemotherapy, we investigated the prognostic value of baseline HRQoL in our study population. Previous clinical trials and real-life studies have demonstrated that baseline HRQoL and subsequent changes during treatment (global and subscales) are associated with survival of patients with pancreatic cancer [8, 9, 11–14, 35]. Our results confirmed this trend in a real-life setting, with patients scoring ≥50 in the QLQ-C30 global health status scale and showing higher OS and PFS.

Remarkably, besides low QLQ-C30 global health status scores at treatment start, low baseline PS was significantly associated with a greater improvement in HRQoL throughout the treatment, presumably because of the greater room for improvement in these patients. Thus, although QLQ-C30 global health status scores at baseline were lower in patients with poorer PS (18- and 21-point differences compared to patients with better PS for KI and ECOG, respectively), PS scores of both groups of patients were consistent after 2 months of chemotherapy. This finding encourages priority assessment of HRQoL in patients with poorer PS. Regarding treatment response, patients with stable disease, or partial or complete response had persistently higher QLQ-C30 global health status scores than those who progressed during the first 3 months of treatment. Of note, HRQoL assessment was restricted to patients receiving first-line treatment, thus gradually reducing the number of patients in the progression subgroup. The observed trend was consistent with data from clinical trials, indicating a relationship between HRQoL and disease progression [3, 5, 6].

In addition to the general limitations of observational designs, such as the uneven sample size across variables due to missing data, the results of this study must be interpreted in the context of the risk of bias associated with a decrease in the study sample over time. This limitation, also observed in previous studies assessing HRQoL [6], implies that patients who discontinue treatment because of disease progression or death—and, therefore, are likely to have poorer HRQoL—are not followed up any longer. Consequently, the study population is gradually biased towards a better HRQoL as the follow-up progresses. Nevertheless, our overall purpose was to describe the changes in HRQoL during first-line chemotherapy, making it necessary to interrupt follow-up in patients initiating second-line chemotherapy. Future studies investigating the evolution of HRQoL throughout further treatment lines shall follow up patients during larger periods, irrespective of the treatment outcome. Furthermore, since a specific questionnaire for assessing HRQoL in patients with pancreatic adenocarcinoma has been validated [36], its use in future studies may be more adequate.

## Conclusions

In summary, our study shows that most patients starting first-line chemotherapy improve their HRQoL throughout the treatment, although this trend might not be applicable to patients who interrupt treatment early in the first few months due to progression or toxicity. Unlike clinical trials, which usually exclude patients with low PS, our study revealed that these patients—which are likely to have poorer HRQoL—may benefit more from chemotherapy in terms of HRQoL. In line with the psychometric properties reported in validation studies of the EORTC QLQ-C30, in our experience, the administration of the questionnaire during routine follow-up visits of patients with mPAC was feasible. Taken together, our results suggest that, in addition to PS, the EORTC QLQ-C30 global health status score may help to identify mPAC patients that are more likely to benefit from chemotherapy., and that HRQoL may be a useful factor to stratify patients in clinical trials. Finally, our study shows that the EORTC QLQ-C30 scale is a responsive tool for identifying changes in HRQoL of mPAC patients who have started first-line chemotherapy.

## Data Availability

Additional data used in this study are available from the corresponding author on reasonable request.

## Abbreviations

mPAC: Metastatic pancreatic adenocarcinoma
HRQoL: Health-related quality of life
PS: Performance status
OS: Overall survival
PFS: Progression free survival
VAS: Visual analog scale
KI: Karnofsky index
